# Influence of slow and rapid weight loss periods on physiological performance, mood state and sleep quality in male freestyle wrestlers: a study from Sichuan Province, China

**DOI:** 10.3389/fpsyg.2024.1445810

**Published:** 2024-10-15

**Authors:** Liang Yu, Lan Lei, Liang Cheng

**Affiliations:** ^1^Human Movement Science, Sichuan Sports College, Chengdu, China; ^2^Sichuan Academy of Chinese Medicine Sciences, Chengdu, China; ^3^School of Sports Medicine and Health, Chengdu Sport University, Chengdu, China

**Keywords:** sports competition, body composition, psychological adaptation, sports training, health impact

## Abstract

**Objective:**

This study aims to investigate the changes in physiological performance, mental state and sleep quality during the weight reduction phase prior to competition in male freestyle wrestlers.

**Methods:**

This study included 16 male freestyle wrestlers from Sichuan Province, China. Participants were evaluated at three time points: the first day of slow weight reduction (T1, March 26), the first day of rapid weight reduction (T2, April 26) and the day before the competition (T3, May 3), and measurements were taken for physiological performance, mood state and sleep quality.

**Results:**

The most relevant findings indicated the following: Morning heart rate, creatine kinase and fatigue scores increased by 12.6, 64.6, and 19.0%, respectively, from T1 to T2 (*p* < 0.05), and decreased by 14.1, 36.6, and 16.8%, respectively, from T2 to T3 (*p* < 0.05). Hemoglobin and testosterone levels decreased by 3.0 and 16.8%, respectively, from T1 to T3 (*p* < 0.05), and by 2.9 and 18.2%, respectively, from T2 to T3 (*p* < 0.05); The secondary findings revealed the following: The total mood disturbance scores decreased by 3.8% from T2 to T3 (*p* = 0.072), positive mood scores decreased by 9.0% from T1 to T2 (*p* = 0.090), the Pittsburgh Sleep Quality Index total scores increased by 14.4% from T1 to T2 (*p* = 0.323) and total work output and average power output decreased by 7.3 and 7.8%, respectively, from T1 to T3 (*p* = 0.067, *p* = 0.052); Regression analyses predicted negative mood (*Y*_1_ = 62.306–0.018 × maximum power output) and sleep quality (*Y*_2_ = 2.527 + 0.278 × Immunoglobulin G) during the weight reduction period.

**Conclusion:**

The combined slow and rapid weight reduction approach effectively minimized body fat in athletes with limited effect on their physiological performance and sleep quality. However, the effects were adverse on certain health variables and anaerobic power in Chinese male freestyle wrestlers. The identified correlations between negative mood and maximum power, and between sleep quality and immunoglobulin G, shed new light on factors influencing athletes’ well-being during weight reduction, and underscored the need for careful monitoring of physiological variables in future weight management strategies.

## 1 Introduction

Freestyle wrestling is a highly demanding competitive sport that places significant emphasis on the physical fitness and technical skills of athletes (Deliceoğlu et al., 2023; Gierczuk and Wójcik, 2023). Athletes often face the challenge of weight class restrictions prior to competitions (Amawi et al., 2024; Hostrup et al., 2024). To meet competition requirements, athletes reduce their body weight to conform to the weight criteria (Reale et al., 2017; Alwan et al., 2022). Traditional rapid weight loss methods, such as strict dietary control and dehydration are effective in the short term but often have negative effects on athletes’ health and competitive performance. These effects include decreased cardiovascular function, weakened muscle strength, impaired immune function, fluctuations in mood state and effects on sleep quality (Garthe et al., 2011; Mendes et al., 2013; Çolak et al., 2020; Lakicevic et al., 2020). A recent systematic review highlights that rapid weight loss in judo athletes, particularly when exceeding 5% per week, significantly increases tension and decreases vigor (Lakicevic et al., 2024). These negative effects may adversely influence athletes’ performance in competitions. Therefore, devising a strategy that can effectively reduce weight with minimal influence on athletes has become an urgent issue in the field of wrestling training.

In a recent comprehensive study by Roklicer et al. (2022a), it was found that the majority of wrestlers, including male freestyle wrestlers, begin weight reduction approximately 7 days prior to competitions. The study also highlighted that the wrestling coach is the most influential person in terms of RWL strategies, and that the methods used significantly differ between wrestling styles. Specifically, for male freestyle wrestlers, the most frequently used methods were increased exercise, gradual dieting, training in a heated room, and sauna (Roklicer et al., 2022a). These findings are particularly relevant to the current study, as they provide insights into the prevalent RWL practices among the population of interest. With the advancement of sports science, the method of combining slow and rapid weight reduction has gradually attracted the attention of wrestlers (Roklicer et al., 2022b; Zholdasbay, 2024). This approach attempts to reduce body fat while maintaining muscle mass through initial slow weight reduction, followed by rapid weight reduction mainly in the short period before the competition to shed water weight and a small amount of fat to reach the competition weight (Martínez-Rodríguez et al., 2021; Baranauskas et al., 2022). This method can balance the weight loss effect and the health status of athletes better. Current research mostly focuses on the influence of weight reduction on athletes’ physical function (Koral and Dosseville, 2009; Yadollahzadeh et al., 2015; Yarar et al., 2019; Ceylan et al., 2022; Mauricio et al., 2022). For instance, rapid weight loss may affect an athlete’s anaerobic capacity in the short term (Yadollahzadeh et al., 2015), and the method and rate of weight loss significantly influences the athlete’s physical functions and competitive state (Yarar et al., 2019). However, studies also consider the effect on athletes’ physical functions to be minimal (Koral and Dosseville, 2009; Ceylan et al., 2022; Mauricio et al., 2022). Research on the mood state and sleep quality of athletes during the weight reduction is rare. A systematic review indicates significant changes in mood states of judo athletes during weight reduction periods (Rossi et al., 2022), whereas another study highlights the influence on sleep behaviors among combat sports athletes during the same period (Dunican et al., 2024). In addition, most studies focus on short-term weight loss effects (Ööpik and Timpmann, 2009; Jouhki et al., 2024; Rhi et al., 2019; Brechney et al., 2022; Samadi et al., 2019) and do not examine athletes’ psychological health and sleep quality during the weight reduction period. These limitations in research have led to a lack of understanding of weight reduction strategies among coaches, especially the in-depth discussion of how weight reduction affects athletes’ psychological stability and sleep quality during the weight reduction period.

While research has been conducted on the impact of weight reduction on athletes’ physical performance, there is a scarcity of literature on its effects on mood state and sleep quality, particularly during the critical pre-competition period. In the realm of freestyle wrestling, the effects of a combined slow and rapid weight reduction strategy on athletes’ psychological and physiological health have not been thoroughly investigated. In light of this, the objective of this study is to provide a scientific basis for the pre-competition weight reduction training of male freestyle wrestlers. By comparing and analyzing the physiological and psychological changes of athletes during the pre-competition weight reduction phases, we aim to offer practical guidance to coaches and athletes. This guidance is intended to assist them in achieving optimal competitive conditions while ensuring their health, thereby enhancing competitive performance and promoting physical well-being. To test the efficacy of this weight reduction strategy, it is hypothesized that there will be significant differences in the changes in physical function, mood state, and sleep quality of male freestyle wrestlers when compared to baseline during both the pre-competition slow weight reduction phase and the rapid weight reduction training period. We anticipate observing a decline in physical function and mood state, along with disruptions to sleep quality, which could potentially affect their competitive performance.

## 2 Materials and methods

### 2.1 Participants

This is an observational cohort study. This study is registered with ClinicalTrials.gov (NCT06543017). This study was approved by the Ethics Committee for Human Experiments at Sichuan Sports College (Number: 2024.4). The research adhered to the Declaration of Helsinki, and all participants were informed of the study’s purpose and intent, and signed informed consent forms. The participants of the study were 16 male freestyle wrestlers from Sichuan Province, China, preparing for the 2024 Chinese Wrestling Championships (4–12th May 2024). Based on their competition categories, they were to participate in five weight classes (57, 61, 65, 70, and 74 kg), with a total of 16 individuals (four in the 70 kg class and three in each of the other classes). The participants had an average age of 20.2 ± 3.2 years, height of 175.4 ± 6.5 cm, weight of 71.1 ± 8.0 kg, body fat percentage of 11.2 ± 1.4% and training experience of 6.9 ± 3.3 years (Table 1). All participants had previously ranked in the top 16 in the annual Chinese freestyle wrestling competitions, which indicates their extensive competitive experience and high level of athletic performance.

**Table 1:** Basic information of participants.

*N*	16
Age (years)	20.2 ± 3.2
Height (cm)	175.4 ± 6.5
Weight (kg)	71.1 ± 8.0
Years of Training (years)	6.9 ± 3.3
Amount of weight loss during the slow weight loss period (kg)	2.9 ± 0.7
Amount of weight loss during the rapid weight loss period (kg)	1.4 ± 0.4

### 2.2 Weight loss management

The study period was 38 days prior to the competition. Based on the training schedule and weight reduction characteristics, the study period was divided into a slow weight reduction phase (where athletes reduced weight slowly by adjusting their dietary structure and increasing the volume of aerobic exercise) and a rapid weight reduction phase (where athletes achieved rapid weight loss by further restricting water intake and adjusting their diet). The slow weight reduction phase was 30 days long (26th March to 25th April 2024), the rapid weight reduction phase was 7 days long (26th April to 2nd May 2024) and the day before the competition was set for the final assessment (3rd May 2024). Slow Weight Loss Phase: Adjust your diet by increasing the intake of vegetables and fruits, and opt for foods high in protein and low in fat; increase your aerobic exercise by jogging or cycling, once a day for 30–60 min; control your total calorie intake by calculating your daily needs based on your basal metabolic rate and activity level, and slightly reduce it to create a mild calorie deficit. Rapid Weight Loss Phase: Further restrict water intake: Under professional guidance, moderately reduce water intake to avoid dehydration while ensuring normal body function; adjust your diet by increasing dietary fiber intake while reducing total calorie intake; engage in high-intensity interval training, once a day for 20–30 min; use a sauna 1–2 times a day for 30 min each session, and replenish lost electrolytes to prevent dehydration.

Three measurements were conducted on all participants, each time between 6:00 and 9:00 in the morning. The first measurement (first day of slow weight reduction, T1) was on 26th March, the second measurement (first day of rapid weight reduction, T2) was on 26th April and the third measurement (the day before the competition, T3) was on 3rd May.

### 2.3 Measurement of indicators

Participants’ heart rate and blood pressure were measured while in a supine resting state, followed by fasting venous blood collection for blood testing, then body composition and questionnaire assessments and finally anaerobic power testing.

#### 2.3.1 Heart rate and blood pressure measurements

Participants’ heart rate and blood pressure were measured in a supine position using an instrument (manufactured in Shenzhen, China, model: CK-W355) by laboratory personnel, and the average of three readings was taken.

#### 2.3.2 Blood measurements

Approximately 3 mL of antecubital venous blood was collected from the participants and evaluated for serum testosterone (Beckman Coulter, United States, chemiluminescence analyzer, Access2), creatine kinase, blood urea (Hitachi, Japan, autoanalyzer, 7180), and immunoglobulin G, immunoglobulin A and immunoglobulin M (Beckman Coulter, United States, protein analyzer, IMMAGE800).

#### 2.3.3 Body composition measurements

Korean Inbody 3.0 body composition analyzer was used to test participants’ body weight and body fat percentage.

#### 2.3.4 Psychological assessments

The Profile of Mood States, a tool for studying mood states and their relation to exercise performance, was used. Participants answered 40 questions with a five-point scale (0–4 points, where 0 indicates “almost none,” 1 “a little,” 2 “moderate,” 3 “quite a bit,” and 4 “extremely”), including seven mood dimensions: tension, anger, fatigue, depression, energy, confusion, and self-esteem. Energy and self-esteem are positive mood dimensions, and higher scores indicating better mood states, whereas the other five are negative mood dimensions, and higher scores indicating worse mood states. The total score, total mood disturbance (calculated as the sum of the five negative mood scores minus the sum of the two positive mood scores plus 100), indicates worse mood states with higher scores (Yu and Cheng, 2024). The scale has a reliability between 0.60 and 0.82 and a validity greater than 0.90 for Chinese individuals (Zhu, 1995); Sleep quality: The Pittsburgh Sleep Quality Index, a questionnaire for assessing sleep quality over the past month, consists of 18 self-rated items forming seven components, with a total score ranging from 0 to 21, where higher scores indicate worse sleep quality. The scale has a reliability between 0.65 and 0.84 and a validity greater than 0.85 for Chinese individuals (Cheng et al., 2023; Tsai et al., 2005).

#### 2.3.5 Anaerobic power measurements

The Wingate 30-s anaerobic test (MONARK 894E, MONARK Company, Sweden) was used. Participants pedaled as fast as possible, adjusted to the prescribed resistance load within 3–4 s and started timing for a 30-s all-out cycling exercise. The average power over 30 s, total work output, maximum power output within 5 s and minimum power output within 5 s were recorded (Nara et al., 2022). The test was stopped if the participant felt unwell during the exercise.

### 2.4 Statistical analysis

Considering the limited number of male freestyle wrestlers in Sichuan Province, China, this study included all available athletes as subjects, resulting in a relatively small sample size. This is similar to previous studies with small samples, all of which were specifically focused on the weight loss procedures and combat sports athletes (Hall and Lane, 2001; Filaire et al., 2001; Reljic et al., 2015; Matthews and Nicholas, 2017). All measured data were processed for mean ± standard deviation using SPSS 19.0. The experimental design followed a 1 (group) × 3 (test times) layout. Data normality was assessed with the Shapiro–Wilk test, and the homogeneity of variance was examined with Mauchly’s Test of Sphericity, which is applicable for both normally and non-normally distributed data. For continuous variables that met the assumptions of normality and homogeneity of variance, a Repeated Measures ANOVA (RM ANOVA) was used to compare measurements across different time points. However, when the assumptions of normality and homogeneity of variance were not satisfied, the non-parametric Friedman test was used to assess differences in both continuous and categorical variables. Post-hoc comparisons for significant RM ANOVA results were conducted using the Bonferroni method to control the overall Type I error rate at a maximum of 0.05 (Cheng et al., 2024; Chang et al., 2024). This study primarily compared differences at various time points, and the effect size was eta-squared (η^2^), where an eta-squared value of η^2^ ≤ 0.01 indicates a small effect, 0.01<η^2^<0.06 indicates a medium effect and η^2^ ≥ 0.06 indicates a large effect (Cohen, 1988). Finally, the overall data measured at the three time points were selected, with negative mood or total sleep score as the dependent variable, and other indicators as independent variables, to establish a multiple regression model. This model was then used to predict the relationship between negative mood or sleep quality and other factors during the weight reduction period in wrestlers. The level of significance was set at *α* = 0.05. To quantify the changes in measured variables, we employed the following formulas to calculate the percentage increase and decrease, which are standard methods described in statistical literature (Zar, 2010). Increase percentage: [(Final Value−Initial Value)/Initial Value] × 100%, which provides a clear representation of the relative increase from the baseline value. Decrease percentage: [(Initial Value−Final Value)/Initial Value] × 100%, which represents the relative reduction from the baseline value.

## 3 Results

Participant weight loss averaged 2.9 kg during the slow reduction period and 1.4 kg during the rapid reduction period. Weight data at three time points (T1: start of slow reduction, T2: start of rapid reduction, T3: day before competition) are in Tables 2–5. Normal distribution and variance homogeneity were confirmed for all variables except serum creatine kinase by the Shapiro–Wilk and Mauchly’s Test of Sphericity.

**Table 2:** Changes in participants’ morning heart rate, blood pressure, and body composition at different time points.

Indicator	T1	T2	T3	*F*	*p*	η^2^
Morning heart rate (beats/min)	49.9 ± 7.3	56.2 ± 8.9*	48.3 ± 6.5^#^	4.930	**0.014**	0.247
Systolic blood pressure (mmHg)	114.2 ± 9.5	116.7 ± 8.7	112.0 ± 8.2	1.311	0.284	0.080
Diastolic blood pressure (mmHg)	63.0 ± 8.5	65.8 ± 9.8	65.7 ± 6.9	0.939	0.402	0.059
Body weight (kg)	71.1 ± 8.0	68.2 + 8.2	66.8 ± 8.1	1.315	0.284	0.081
Body fat percentage (%)	11.2 ± 1.4	8.8 ± 1.1	8.5 ± 1.1	0.684	0.512	0.044

T1: First day of slow weight reduction; T2: First day of rapid weight reduction; T3: Day before the competition; F, P, η^2^: Subject effect test at 3 time points; Compared with T1, T2 shows a significant difference at * indicating *p* < 0.05; compared with T2, T3 shows a significant difference at # indicating *p* < 0.05. Bold values indicate statistical significance.

**Table 3:** Changes in participants’ blood indicators at different time points.

Indicator	T1	T2	T3	*F*	*p*	η^2^
Hemoglobin (g/L)	161.7 ± 6.0	161.5 ± 4.8	156.8 ± 4.6^#&^	5.040	**0.013**	0.251
Serum creatine kinase (U/L)	252.2 ± 61.0	415.1 ± 163.2**	263.2 ± 96.9^#^	9.250	**0.001**	0.381
Blood urea (mmol/L)	6.96 ± 1.20	7.31 ± 1.10	7.37 ± 1.08	0.506	0.608	0.033
Serum testosterone (ng/dL)	641.8 ± 90.7	652.7 ± 106.5	533.7 ± 98.1^##&^	7.302	0.003	0.327
White blood cell count (10^9^/L)	5.95 ± 0.82	6.46 ± 1.40	6.11 ± 1.12	0.859	0.434	0.054
Immunoglobulin G (g/L)	10.66 ± 1.90	10.87 ± 2.09	10.54 ± 1.63	0.106	0.900	0.007
Immunoglobulin A (g/L)	2.40 ± 0.56	2.36 ± 0.53	2.32 ± 0.48	0.111	0.895	0.007
Immunoglobulin M (g/L)	1.36 ± 0.52	1.33 ± 0.53	1.30 ± 0.50	0.068	0.934	0.005

T1: First day of slow weight reduction; T2: First day of rapid weight reduction; T3: Day before the competition; F, P, η^2^: Subject effect test at 3 time points; Compared with T1, T2 has ** indicating *p* < 0.01; compared with T2, T3 has # indicating *p* < 0.05 and ## indicating *p* < 0.01; compared with T1, T3 has & indicating *p* < 0.05. Bold values indicate statistical significance.

**Table 4:** Changes in participants’ psychological assessments at different time points.

Indicator	T1	T2	T3	*F*	*p*	η^2^
Tension scores	11.9 ± 3.0	11.0 ± 3.3	10.5 ± 2.5	0.820	0.450	0.052
Anger scores	10.6 ± 2.6	9.7 ± 3.0	10.3 ± 3.4	0.391	0.680	0.025
Fatigue scores	10.0 ± 2.0	11.9 ± 2.6*	9.9 ± 2.9^#^	**3.577**	**0.040**	0.193
Depression scores	7.5 ± 2.3	8.2 ± 2.5	8.0 ± 2.3	0.693	0.508	0.044
Confusion scores	8.7 ± 2.7	8.3 ± 1.9	8.0 ± 2.7	1.004	0.378	0.063
Energy scores	18.3 ± 3.1	16.8 ± 3.9	18.5 ± 3.5	1.020	0.373	0.064
Self-esteem scores	14.1 ± 4.5	12.7 ± 2.9	13.2 ± 3.0	0.578	0.567	0.037
Total mood disturbance scores	116.3 ± 14.8	119.6 ± 14.0	115.0 ± 14.8	2.007	0.152	0.118
Negative mood scores	48.7 ± 8.8	49.1 ± 10.8	46.7 ± 11.1	1.057	0.360	0.066
Positive mood scores	32.4 ± 7.0	29.5 ± 6.4	31.7 ± 6.5	1.961	0.158	0.116
PSQI scores	5.15 ± 1.83	5.89 ± 1.60	5.52 ± 1.71	0.749	0.482	0.048

T1: First day of slow weight reduction; T2: First day of rapid weight reduction; T3: Day before the competition; F, P, η^2^: Subject effect test at 3 time points; Compared with T1, T2 has a * indicating *p* < 0.05; compared with T2, T3 has a # indicating *p* < 0.05. Bold values indicate statistical significance.

**Table 5:** Changes in participants’ anaerobic power at different time points.

Indicator	T1	T2	T3	*F*	*p*	η^2^
Total work output (W)	3630.9 ± 410.8	3530.2 ± 350.8	3367.0 ± 411.5	2.103	0.140	0.123
Maximum power output (W)	792.1 ± 104.7	763.5 ± 96.3	751.9 ± 95.9	0.770	0.472	0.049
Minimum power output (W)	440.5 ± 73.4	432.7 ± 69.8	403.6 ± 65.7	1.391	0.264	0.085
Average power output (W)	590.4 ± 80.3	578.8 ± 75.2	544.3 ± 72.6	2.606	0.090	0.148

T1: First day of slow weight reduction; T2: First day of rapid weight reduction; T3: Day before the competition; F, P, η^2^: Subject effect test at 3 time points.

Significant changes across time points included: morning heart rate (increased 12.6% from T1 to T2, *p* = 0.022; decreased 14.1% from T2 to T3, *p* = 0.023); creatine kinase (increased 64.6% from T1 to T2, *p* = 0.002; decreased 36.6% from T2 to T3, *p* = 0.015); fatigue (increased 19.0% from T1 to T2, *p* = 0.019; decreased 16.8% from T2 to T3, *p* = 0.040); hemoglobin (decreased 3.0% from T1 to T3, p = 0.019; 2.9% from T2 to T3, *p* = 0.021); testosterone (decreased 16.8% from T1 to T3, *p* = 0.011; 18.2% from T2 to T3, *p* = 0.001).

Non-significant trends included: total mood disturbance score (decreased 3.8% from T2 to T3, *p* = 0.072); positive mood score (decreased 9.0% from T1 to T2, *p* = 0.090); Pittsburgh Sleep Quality Index (increased 14.4% from T1 to T2, *p* = 0.323); total work output (decreased 7.3% from T1 to T3, *p* = 0.067); average power output (decreased 7.8% from T1 to T3, *p* = 0.052).

Regression analyses predicted negative mood (Y_1_ = 62.306–0.018 × maximum power output; *F*_(1, 46)_ = 4.907, *p* = 0.032, *R*^2^ = 0.077) and sleep quality (Y_2_ = 2.527 + 0.278 × Immunoglobulin G; *F*_(1, 46)_ = 4.859, *p* = 0.033, *R*^2^ = 0.096) during the weight reduction period.

## 4 Discussion

This study aimed to explore the changes in physiological function, mood state and sleep quality in male freestyle wrestlers during the precompetition period utilizing a strategy that combines slow and rapid weight reduction. This study provides a scientific basis for the weight reduction training of wrestlers before competitions and offers practical guidance to coaches and athletes to assist them in achieving optimal competitive states while ensuring health.

The hypothesis of this study was partially confirmed. Athletes’ morning heart rate, creatine kinase and fatigue scores significantly increased to varying degrees on the first day of rapid weight reduction compared with the first day of slow weight reduction. However, these parameters significantly decreased to varying degrees on the day before the competition compared with the first day of rapid weight reduction. Hemoglobin and testosterone levels also significantly decreased to varying degrees on the day before the competition compared with the first day of slow weight reduction. A study on 18 judo athletes undergoing precompetition weight reduction found a significant increase in creatine kinase and suggests that precompetition weight reduction may accelerate muscle damage during training, accompanied by a decrease in hemoglobin and testosterone (Roklicer et al., 2020). Examinations of endurance athletes revealed that an increase in morning heart rate was related to increased training load and levels of serum markers of muscle strain (creatine kinase) (Weippert et al., 2018). A systematic review of 1,103 judo athletes undergoing precompetition weight reduction found that feelings of tension, anger, and fatigue significantly increased in athletes undergoing rapid weight loss (Lakicevic et al., 2024). Consistent with our hypothesis, this study partially supports previous findings (Weippert et al., 2018; Lakicevic et al., 2020; Roklicer et al., 2020) and highlights the substantial influence of weight reduction on athletes’ physiological and psychological well-being. Notably, the distinct phases of slow and rapid weight reduction exerted varying degrees of influence and underscored the need for tailored strategies to mitigate potential adverse effects on athletes’ performance and recovery.

Possible reasons for the changes in morning heart rate, creatine kinase and fatigue scores may be influenced by various factors. For instance, the increase in morning heart rate may be related to the increased activity of the sympathetic nervous system during rapid weight reduction and reflects the body’s stress response to increased energy expenditure (Harrell et al., 2016). Recent research also emphasizes the negative influence of rapid weight loss on heart rate recovery in Greco–Roman wrestlers (Roklicer et al., 2022a). The increased morning heart rate observed in our study may be related to the phenomenon of slowed heart rate recovery reported by the study (Roklicer et al., 2022a). This finding suggests that rapid weight loss strategies may interfere with athletes’ recovery ability by affecting the regulation of the autonomic nervous system, which is consistent with the increased fatigue and decreased performance indicators that we observed. Over time, athletes may reduce their morning heart rate by improving cardiovascular efficiency and metabolic adaptability, thus demonstrating the body’s adaptability to weight reduction strategies. The significant changes in creatine kinase may be related to muscle microinjury and subsequent repair processes during weight reduction (Cheng et al., 2022), which may be influenced by training intensity, recovery strategies and nutritional intake, and increases potentially enhance athletes’ fatigue performance. The decrease in hemoglobin and testosterone levels may be related to various biological mechanisms. Rapid weight loss may lead to a decrease in blood volume and affect the transport efficiency of hemoglobin. Additionally, reduced energy intake may affect the availability of precursor substances for hormone synthesis, thereby influencing the production of testosterone (Grossmann and Wittert, 2021). These hormonal changes may indirectly affect athletes’ performance and recovery ability by influencing energy metabolism, muscle synthesis and mood state.

Additionally, this study developed multiple regression models, utilizing negative mood and total sleep scores as dependent variables, alongside various other indicators as independent variables. This analysis aimed to forecast how negative mood and sleep quality are associated with other factors throughout the weight reduction phase in wrestlers. The model for negative mood was defined as Y_1_ = 62.306–0.018 × maximum power output, whereas the model for sleep quality was expressed as Y_2_ = 2.527 + 0.278 × Immunoglobulin G. Research has demonstrated a link between athletes’ negative emotions and their performance (Lazarus, 2000), yet the interplay between sleep quality and immunoglobulin G levels remains understudied. Existing literature indicates that higher serum immunoglobulin G levels can be associated with sleep deprivation (Abbasmanesh et al., 2019), overtraining (MacKinnon, 2000) and suboptimal athletic performance periods (Solomon and Weiss Kelly, 2016). Our findings, which highlight the correlations between negative emotions and maximum power output, as well as sleep quality and immunoglobulin G, contribute to the existing body of knowledge. These insights offer a novel perspective on the factors influencing the psychological and sleep aspects of athletes’ well-being during weight reduction. This research could inform future investigations exploring similar dynamics. A study highlights the critical gaps in combat sports athletes’ knowledge and practices regarding sleep behaviors, shift work disorders, alcohol consumption and nutritional knowledge, which aligns with the changes in mood state and sleep quality observed in athletes during the weight reduction period (Dunican et al., 2024).

The correlation between negative emotions and maximum power output may reflect the direct effect of mood state on athletic performance. High training loads may lead to increased fatigue and stress (Hamlin et al., 2019), thereby increasing negative emotions. The correlation between sleep quality and immunoglobulin G may indicate the role of sleep in maintaining immune system function and promoting physical recovery. These findings emphasize the importance of monitoring and managing the mood state and sleep quality of athletes during the weight reduction period.

Although participants’ total mood disturbance scores, positive mood scores, Pittsburgh Sleep Quality Index scores, total work output and average power output showed clear trends at different time points, they did not reach statistical significance. A systematic review indicates that judo athletes experience significant changes in mood states during weight reduction periods, including increased negative emotions and decreased vigor, and an overall increase in TMD scores (Rossi et al., 2022). By contrast, this study indicates that the combination of slow and rapid weight reduction methods helped athletes meet weight requirements without significant decreases in cardiovascular function, which indicates satisfactory physiological adaptability. In terms of mood state, although fatigue increased during the slow weight reduction period, it was alleviated after rapid weight reduction, and other negative emotions did not significantly increase, which indicates that athletes could adapt to the stress (Huh, 2018) during the weight reduction period. Sleep quality remained stable throughout the weight reduction period, which indicates that athletes’ sleep was not significantly adversely affected by the weight reduction strategy. However, these nonsignificant trends could be attributed to factors such as small sample size, individual differences, variability in individual adaptability to weight reduction strategies or measurement errors. Future research should account for these potential confounding factors and verify these trends with larger samples and more precise measurement techniques.

## 5 Study limitations

This study has certain limitations. Firstly, due to the use of convenience sampling, the results may be affected by sample selection bias and may not be entirely applicable to wrestlers from other regions or of different skill levels. Future research will consider using random sampling or other more representative sampling methods to understand the physiological and psychological changes better in different populations during the weight reduction period. Secondly, the duration of the study is short, focuses on changes during the precompetition weight reduction period and has no long-term follow-up of the athletes’ health and competitive performance. Future research should expand the sample size to include athletes of different sports and extend the study duration to evaluate the long-term effects of weight reduction strategies more comprehensively. Additionally, this study relies primarily on the athletes’ self-reports when assessing mood state and sleep quality, which may be subject to subjective influences. Future studies could combine objective physiological indicators and behavioral observations to improve the accuracy of assessments.

## 6 Conclusion

This study supports the combination of slow and rapid weight reduction as an effective precompetition weight reduction strategy, which minimizes the negative effect on athletes’ physical function and mood state while reducing body weight and fat content. Although the trends in indicators such as mood, Pittsburgh Sleep Quality Index total scores, total work output and average power output did not reach statistical significance, these trends suggest that monitoring these psychological and physiological indicators during the weight reduction period may be necessary. The multiple regression models revealed correlations between negative emotions and maximum power output, and between sleep quality and immunoglobulin G, thus providing a new perspective for understanding the factors affecting athletes’ mood state and sleep quality during the weight reduction period. However, weight reduction indeed had adverse effects on certain health-related variables and anaerobic power parameters of Chinese male freestyle wrestlers, which warrants further exploration and resolution in future research.

## Data Availability

The raw data supporting the conclusions of this article will be made available by the authors, without undue reservation.

## References

[ref1] Abbasmanesh M. Shetabboushehri N. Zarghmi M. (2019). Effect of sleep deprivation on mood and reaction time in the athletes and non-athletes. Rooyesh 8, 55–62.

[ref2] Alwan N. Moss S. L. Davies I. G. Elliott-Sale K. J. Enright K. (2022). Weight loss practices and eating behaviours among female physique athletes: acquiring the optimal body composition for competition. PLoS One 17:e0262514. doi: 10.1371/journal.pone.0262514, PMID: 35030218 PMC8759685

[ref3] Amawi A. AlKasasbeh W. Jaradat M. Almasri A. Alobaidi S. Hammad A. A.. (2024). Athletes’ nutritional demands: a narrative review of nutritional requirements. Front. Nutr. 10:1331854. doi: 10.3389/fnut.2023.1331854, PMID: 38328685 PMC10848936

[ref4] Baranauskas M. Kupčiūnaitė I. Stukas R. (2022). The association between rapid weight loss and body composition in elite combat sports athletes. Healthcare 10:665. doi: 10.3390/healthcare10040665, PMID: 35455842 PMC9031560

[ref5] Brechney G. C. Cannon J. Goodman S. P. (2022). Effects of weight cutting on exercise performance in combat athletes: a meta-analysis. Int. J. Sports Physiol. Perform. 17, 995–1010. doi: 10.1123/ijspp.2021-0104, PMID: 35523423

[ref6] Ceylan B. Aydos L. Šimenko J. (2022). Effect of rapid weight loss on hydration status and performance in elite judo athletes. Biology 11:500. doi: 10.3390/biology11040500, PMID: 35453700 PMC9031997

[ref7] Chang S. W. Cheng L. Liu H. (2024). Effects of three-duration tai-chi exercises on depression and sleep quality in older women. Eur. Geriatr. Med. 15, 1141–1148. doi: 10.1007/s41999-024-00981-4, PMID: 38693298

[ref8] Cheng L. Chang S. W. Wang B. C. He B. X. Tan Y. J. (2023). Cross- sectional study of depression tendency and sleep quality in 1352 people practicing tai chi. Res. Sports Med. 31, 650–662. doi: 10.1080/15438627.2021.2024832, PMID: 34994259

[ref9] Cheng L. Wang K. Chang S. W. Tan Y. J. He B. X. (2024). Effects of platelet-rich plasma combined with isometric quadriceps contraction on cartilage in a rat model of knee osteoarthritis. Regen. Ther. 26, 469–477. doi: 10.1016/j.reth.2024.06.021 39070125 PMC11283084

[ref10] Cheng L. Wang K. He B. Yan Y. (2022). Effect of vertical vibration stimulation at different frequencies on delayed muscle soreness in athletes: a randomized trial. Front. Public Health 10:980454. doi: 10.3389/fpubh.2022.980454, PMID: 36311634 PMC9614366

[ref11] Cohen J. (1988). Statistical power analysis for the behavioral sciences. New York, NY: Routledge Academic.

[ref12] Çolak A. Şahin İ. Soylu Y. Koç M. Ocal T. (2020). Weight loss methods and effects on the different combat sports athletes. Prog. Nutr. 22, 119–124. doi: 10.23751/pn.v22i1-S.9803

[ref13] Deliceoğlu G. Tortu E. Keleş A. Çakar A. N. Özsoy H. Ö. Atalay T. A. (2023). Comparison of physical and physiological profiles between elite freestyle men and women wrestlers. Eur. J. Hum. Mov. 50, 103–124. doi: 10.21134/eurjhm.2023.50.11

[ref14] Dunican I. C. Galpin A. Turner M. Reale R. (2024). Sleep behaviors and nutritional knowledge in amateur and professional combat sport athletes. J. Strength Cond. Res. 38, 1627–1634. doi: 10.1519/JSC.0000000000004846, PMID: 38985931

[ref15] Filaire E. Maso F. Degoutte F. Jouanel P. Lac G. (2001). Food restriction, performance, psychological state and lipid values in judo athletes. Int. J. Sports Med. 22, 454–459. doi: 10.1055/s-2001-16244, PMID: 11531040

[ref16] Garthe I. Raastad T. Sundgot-Borgen J. (2011). Long-term effect of weight loss on body composition and performance in elite athletes. Int. J. Sport Nutr. Exerc. Metab. 21, 426–435. doi: 10.1123/ijsnem.21.5.426 21896944

[ref17] Gierczuk D. Wójcik Z. (2023). Physical fitness of highly qualified female and male wrestlers of various sports levels. J. Phys. Educ. Sport 23, 1488–1494. doi: 10.7752/jpes.2023.06182

[ref18] Grossmann M. Wittert G. A. (2021). Dysregulation of the hypothalamic–pituitary– testicular axis due to energy deficit. J. Clin. Endocrinol. Metab. 106, 4861–4871. doi: 10.1210/clinem/dgab517 34264314

[ref19] Hall C. J. Lane A. M. (2001). Effects of rapid weight loss on mood and performance among amateur boxers. Br. J. Sports Med. 35, 390–395. doi: 10.1136/bjsm.35.6.390, PMID: 11726472 PMC1724425

[ref20] Hamlin M. J. Wilkes D. Elliot C. A. Lizamore C. A. Kathiravel Y. (2019). Monitoring training loads and perceived stress in young elite university athletes. Front. Physiol. 10:34. doi: 10.3389/fphys.2019.00034, PMID: 30761016 PMC6361803

[ref21] Harrell C. S. Gillespie C. F. Neigh G. N. (2016). Energetic stress: the reciprocal relationship between energy availability and the stress response. Physiol. Behav. 166, 43–55. doi: 10.1016/j.physbeh.2015.10.009 26454211 PMC4826641

[ref22] Hostrup M. Hansen E. S. Rasmussen S. M. Jessen S. Backer V. (2024). Asthma and exercise-induced bronchoconstriction in athletes: diagnosis, treatment, and anti-doping challenges. Scand. J. Med. Sci. Sports 34:e14358. doi: 10.1111/sms.14358, PMID: 36965010

[ref23] Huh Y. S. (2018). Effects of short-term weight loss on stress hormone and EEG in weight athletes. Exer Sci 27, 146–152. doi: 10.15857/ksep.2018.27.2.146

[ref24] Jouhki I. Sarin H. V. Jauhiainen M. O’Connell T. M. Isola V. Ahtiainen J. P.. (2024). Effects of fat loss and low energy availability on the serum cardiometabolic profile of physique athletes. Scand. J. Med. Sci. Sports 34:e14553. doi: 10.1111/sms.14553, PMID: 38268074

[ref25] Koral J. Dosseville F. (2009). Combination of gradual and rapid weight loss: effects on physical performance and psychological state of elite judo athletes. J. Sports Sci. 27, 115–120. doi: 10.1080/02640410802413214, PMID: 19058087

[ref26] Lakicevic N. Roklicer R. Bianco A. Mani D. Paoli A. Trivic T.. (2020). Effects of rapid weight loss on judo athletes: a systematic review. Nutrients 12:1220. doi: 10.3390/nu12051220 32357500 PMC7281976

[ref27] Lakicevic N. Thomas E. Isacco L. Tcymbal A. Pettersson S. Roklicer R.. (2024). Rapid weight loss and mood states in judo athletes: a systematic review. Eur. Rev. Appl. Psychol. 74:100933. doi: 10.1016/j.erap.2023.100933

[ref28] Lazarus R. S. (2000). How emotions influence performance in competitive sports. Sport Psychol. 14, 229–252. doi: 10.1123/tsp.14.3.229

[ref29] MacKinnon L. T. (2000). Overtraining effects on immunity and performance in athletes. Immunol. Cell Biol. 78, 502–509. doi: 10.1111/j.1440-1711.2000.t01-7-.x 11050533

[ref30] Martínez-Rodríguez A. Vicente-Salar N. Montero-Carretero C. Cervelló-Gimeno E. Roche E. (2021). Weight loss strategies in male competitors of combat sport disciplines. Medicina 57:897. doi: 10.3390/medicina57090897, PMID: 34577820 PMC8467103

[ref31] Matthews J. J. Nicholas C. (2017). Extreme rapid weight loss and rapid weight gain observed in UK mixed martial arts athletes preparing for competition. Int. J. Sport Nutr. Exerc. Metab. 27, 122–129. doi: 10.1123/ijsnem.2016-0174, PMID: 27710145

[ref32] Mauricio C. D. A. Merino P. Merlo R. Vargas J. J. N. Chávez J. Á. R. Pérez D. V.. (2022). Rapid weight loss of up to five percent of the body mass in less than 7 days does not affect physical performance in official Olympic combat athletes with weight classes: a systematic review with meta-analysis. Front. Physiol. 13:830229. doi: 10.3389/fphys.2022.830229, PMID: 35492609 PMC9039236

[ref33] Mendes S. H. Tritto A. C. Guilherme J. P. L. Solis M. Y. Vieira D. E. Artioli G. G. (2013). Effect of rapid weight loss on performance in combat sport male athletes: does adaptation to chronic weight cycling play a role? Br. J. Sports Med. 47, 1155–1160. doi: 10.1136/bjsports-2013-092689, PMID: 24047570

[ref34] Nara K. Kumar P. Rathee R. Kumar J. (2022). The compatibility of running-based anaerobic sprint test and Wingate anaerobic test: a systematic review and meta-analysis. Pedagogy Phys. Cult. Sports 26, 134–143. doi: 10.15561/26649837.2022.0208

[ref35] Ööpik V. Timpmann S. (2009). Weight loss and physical performance capacity in combat sports athletes: impact of nutritional factors. Sporto Mokslas 1, 33–39.

[ref36] Reale R. Slater G. Burke L. M. (2017). Individualised dietary strategies for Olympic combat sports: acute weight loss, recovery and competition nutrition. Eur. J. Sport Sci. 17, 727–740. doi: 10.1080/17461391.2017.1297489, PMID: 28316263

[ref37] Reljic D. Jost J. Dickau K. Kinscherf R. Bonaterra G. Friedmann B. B. (2015). Effects of pre-competitional rapid weight loss on nutrition, vitamin status and oxidative stress in elite boxers. J. Sports Sci. 33, 437–448. doi: 10.1080/02640414.2014.949825 25259507

[ref38] Rhi S. Lee J. Lee Y. Kim K. (2019). Changes of body composition, physical fitness, and blood variables according to the short-term weight reduction in college ssireum athletes. J. Exerc. Rehabil. 15, 74–77. doi: 10.12965/jer.1836526.263, PMID: 30899740 PMC6416499

[ref39] Roklicer R. Lakicevic N. Stajer V. Trivic T. Bianco A. Mani D.. (2020). The effects of rapid weight loss on skeletal muscle in judo athletes. J. Transl. Med. 18, 142–147. doi: 10.1186/s12967-020-02315-x, PMID: 32228627 PMC7106841

[ref40] Roklicer R. Rossi C. Bianco A. Stajer V. Ranisavljev M. Drid P. (2022a). Prevalence of rapid weight loss in Olympic style wrestlers. J. Int. Soc. Sports Nutr. 19, 593–602. doi: 10.1080/15502783.2022.2119095 36250149 PMC9559051

[ref9001] Roklicer R. Rossi C. Bianco A. Stajer V. Maksimovic N. Manojlovic M.. (2022b). Rapid weight loss coupled with sport-specific training impairs heart rate recovery in greco-roman wrestlers. Appl. Sci. 12:3286. doi: 10.3390/app12073286

[ref41] Rossi C. Roklicer R. Tubic T. Bianco A. Gentile A. Drid P. (2022). The role of psychological factors in judo: a systematic review. Int. J. Environ. Res. Public Health 19:2093. doi: 10.3390/ijerph19042093 35206281 PMC8871700

[ref42] Samadi M. Chaghazardi M. Bagheri A. Karimi S. Pasdar Y. Moradi S. (2019). A review of high-risk rapid weight loss behaviors with assessment of food intake and anthropometric measurements in combat sport athletes. Asian J. Sports Med. 10:e85697. doi: 10.5812/asjsm.85697

[ref43] Solomon M. L. Weiss Kelly A. K. (2016). Approach to the underperforming athlete. Pediatr. Ann. 45, 91–96. doi: 10.3928/00904481-20160210-02 27031317

[ref44] Tsai P. S. Wang S. Y. Wang M. Y. Su C. T. Yang T. T. Fang S. C. (2005). Psychometric evaluation of the Chinese version of the Pittsburgh sleep quality index (CPSQI) in primary insomnia and control subjects. Qual. Life Res. 14, 1943–1952. doi: 10.1007/s11136-005-4346-x 16155782

[ref46] Weippert M. Behrens M. Mau-Moeller A. Bruhn S. Behrens K. (2018). Relationship between morning heart rate variability and creatine kinase response during intensified training in recreational endurance athletes. Front. Physiol. 9:1267. doi: 10.3389/fphys.2018.01267, PMID: 30298014 PMC6161148

[ref47] Yadollahzadeh R. Jourkesh M. Antonio J. Soori R. (2015). The effects of rapid weight loss on aerobic and anaerobic power on athletes in weight-sensitive sports. Sport Sci. 8, 30–34.

[ref48] Yarar H. Türkyilmaz R. Eroğlu H. (2019). The investigation of weight loss profiles on weight classes sports athletes. Int. J. Appl. Exerc. Physiol. 8, 62–68.

[ref49] Yu L. Cheng L. (2024). The work stress, occupational burnout, coping strategies and organizational support of elite sports coaches in Sichuan Province: the mediating role of organizational support. Front. Psychol. 15:1437234. doi: 10.3389/fpsyg.2024.1437234, PMID: 39171218 PMC11335728

[ref50] Zar J. H. (2010). Biostatistical analysis. 5th Edn: Prentice Hall.

[ref51] Zholdasbay A. (2024). Integration of physical and psychological training of wrestlers at the stage of preparation for the elite level. Sports Sci. Health 27, 49–57. doi: 10.7251/SSH24V049Z

[ref52] Zhu B. L. (1995). Brief introduction of POMS scale and its model for China. J. Tianjin Inst. Phys. Educ. 10, 35–37. doi: 10.13297/j.cnki.issn1005-0000.1995.01.007

